# Quantum Attacks on Sum of Even–Mansour Construction with Linear Key Schedules

**DOI:** 10.3390/e24020153

**Published:** 2022-01-20

**Authors:** Ping Zhang

**Affiliations:** School of Computer Science, Nanjing University of Posts and Telecommunications, Nanjing 210023, China; zhgp@njupt.edu.cn

**Keywords:** quantum attacks, sum of Even–Mansour construction, linear key schedule, quantum algorithms

## Abstract

Shinagawa and Iwata are considered quantum security for the sum of Even–Mansour (SoEM) construction and provided quantum key recovery attacks by Simon’s algorithm and Grover’s algorithm. Furthermore, quantum key recovery attacks are also presented for natural generalizations of SoEM. For some variants of SoEM, they found that their quantum attacks are not obvious and left it as an open problem to discuss the security of such constructions. This paper focuses on this open problem and presents a positive response. We provide quantum key recovery attacks against such constructions by quantum algorithms. For natural generalizations of SoEM with linear key schedules, we also present similar quantum key recovery attacks by quantum algorithms (Simon’s algorithm, Grover’s algorithm, and Grover-meet-Simon algorithm).

## 1. Introduction

Since 1981, when Richard Feynman, winner of the Nobel Prize in Physics, proposed the concept of a quantum computer, the research of the quantum computer has deeply influenced the scientific research circle. Classical computers are often attacked by malicious viruses that crash the computer and can lead to personal information being stolen. However, in quantum computers, these problems will not exist because of the quantum no-cloning principle and Heisenberg’s uncertainty principle. Quantum computers have good properties, such as fast running speed, a strong information processing ability, and powerful parallel computing capability. Therefore, quantum computers have great applications in cryptanalysis and other fields. Quantum algorithms are the most important software components of quantum computers to realize quantum computation.

The importance of information security is self-evident. In 2021, there were multiple breaches of sensitive information and cyber attacks, causing a large number of property losses and even endangering personal security and social stability. Modern cryptography is one of the core technologies to protect information security.

The design and analysis of cryptographic schemes that resist quantum computing have become increasingly important. Among them, the public key cryptographic scheme is the typical representative. Difficult mathematical problems of public key cryptography can be solved by efficient quantum algorithms. Therefore, public key cryptographic schemes, such as RSA and ECC, are insecure in the quantum scenario [1]. While, for symmetric cryptographic schemes (such as AES and IDEA), the influence is limited and Grover’s algorithm [2] has been regarded for a long time as the best method to search for the secret key. It is only in recent years that quantum analyses of symmetric cryptographic schemes have made some progress.

Simon’s algorithm [3] is a vital quantum algorithm for the quantum analyses of symmetric cryptographic schemes. Its goal is to efficiently find a period of a period function. It was first utilized to the security analyses of the 3-round Feistel cipher [4] and then extended to the Even–Mansour cipher [5,6], Feistel and its variants [7,8,9,10,11], and the Luby–Rackoff construction [12]. Grover-meet-Simon algorithm [13] was first introduced by Leander and May, and combined Simon’s algorithm and Grover’s algorithm to achieve the key recovery attack against FX-construction. Currently, Simon’s algorithm, Grover’s algorithm, and Grover-meet-Simon algorithm have been extended to the Sum of Even–Mansour construction [14], encryption schemes [15,16,17,18,19,20], hash schemes [21,22,23], message authentication codes (MACs) [18,24], and authenticated encryption schemes [18,25,26]. There exist other quantum algorithms (such as HHL algorithm and BTH algorithm) and relevant quantum cryptanalysis. We will not go into the details here.

**Problem Statement.** The sum of Even–Mansour (SoEM) construction [14] is built by the exclusive or (XOR) of two instances of Even–Mansour cipher. According to whether the keys or permutations used in the two instances are equal, SoEM is divided into three variants: SoEM1 for the case where permutations used in the two instances are identical, SoEM21 for the case where permutations used in the two instances are independent but keys used in the two instances are identical, and SoEM22 for the case where permutations used in the two instances are independent and keys used in the two instances are independent. They are pseudorandom functions designed by random permutations and designers give security results in the classical scenario.

Shinagawa and Iwata considered the quantum security for SoEM construction, providing quantum key recovery attacks by Simon’s algorithm and Grover’s algorithm, and applied the similar quantum key recovery attacks to natural generalizations of SoEM in [27]. For some variants of SoEM, they found that their quantum attacks are not obvious and left it as an open problem to consider the security of such constructions.

**Our Contributions.** This paper focuses on the open problem and provides quantum key recovery attacks against such constructions by quantum algorithms. First, we consider a variant of SoEM21 given in Shinagawa and Iwata, which is described as:

$$\begin{array}{c} C=SoEM21_{K}^{P_{1},P_{2}}\left(M\right)=P_{1}\left(M⊕K\right)⊕K⊕P_{2}\left(M⊕2\cdot K\right)⊕2\cdot K, \end{array}$$

where $P_{1}$ and $P_{2}$ are two public *n*-bit random permutations, *K* is an *n*-bit key, *M* is a plaintext, and *C* is the corresponding ciphertext. Here SoEM21 is generated by the XOR-sum of two instances of Even–Mansour cipher with simple key schedules. We prove that this variant is insecure under the quantum scenario and recover its key by quantum algorithms.

Then we consider a generalized construction of SoEM21 with linear key schedules (a linear key schedule means that it is linear with respect to the key ) and rename it as SoEM21L, which is described as:

$$\begin{array}{c} C=SoEM21L_{K}^{P_{1},P_{2}}\left(M\right)=P_{1}\left(M⊕a\cdot K\right)⊕P_{2}\left(M⊕b\cdot K\right)⊕c\cdot K, \end{array}$$

where a,b,c are three integers and (a,b,c)≠(0,0,0). We also achieve a quantum key recovery attack against SoEM21L by quantum algorithms.

Finally, we consider natural generalizations of SoEM with linear key schedules and present similar quantum key recovery attacks by quantum algorithms (Simon’s algorithm, Grover’s algorithm, and Grover-meet-Simon algorithm).

**Organizations of This Paper.** Notations and some preliminaries are presented in Section 2. Quantum algorithms are shown in Section 3. In Section 4, we describe quantum key recovery attacks for SoEM21 and SoEM21L. In Section 5, we present natural generalizations of SoEM with linear key schedules and their quantum key recovery attacks. Finally, we present a conclusion in Section 6.

## 2. Preliminaries

**Notations.** Given an integer n≥1, let ${\left\{0,1\right\}}^{n}$ be a set of all strings whose bit-lengths are *n*, and Perm(n) be a set of all permutations over ${\left\{0,1\right\}}^{n}$. For any two finite strings $x\in {\left\{0,1\right\}}^{n}$ and $y\in {\left\{0,1\right\}}^{n}$, let x⊕y stand for their bit-wise XOR.

**Finite Field.** The finite field $GF(2^{n})$ can be viewed as the set ${\left\{0,1\right\}}^{n}$ and $GF\left(2^{n}\right)=GF\left(2\right)/\left(f\left(x\right)\right)$, where f(x) is an irreducible polynomial of degree *n* over GF(2). For any integer $0\leq a\leq 2^{n}-1$, it can be seen as an *n*-bit string over $GF(2^{n})$, i.e., $a=a_{n-1}\cdots a_{1}a_{0}\in {\left\{0,1\right\}}^{n}$, where $a_{i}\in \left\{0,1\right\}$ for 0≤i≤n−1. It also corresponds to a polynomial with a degree of at most n−1 over {0,1}, i.e., $a\left(x\right)=a_{n-1}x^{n-1}+\cdots +a_{1}x+a_{0}$. For example, 2 (10) corresponds to *x*, 3 (11) corresponds to x+1, and 7 (111) corresponds to $x^{2}+x+1$. The addition over $GF(2^{n})$ can be defined by the addition of polynomials over {0,1} or the bit-wise XOR over ${\left\{0,1\right\}}^{n}$ and the multiplication over $GF(2^{n})$ is defined by the polynomial multiplication over {0,1} reduced modulo f(x), i.e., for any $a,b\in GF(2^{n})$, then a+b=a(x)+b(x)mod2=a⊕b and a·b=a(x)·b(x) mod f(x). Therefore, if n=128 and $f\left(x\right)=x^{128}+x^{7}+x^{2}+x+1$, then:

$$\begin{array}{cc} \; & 2\cdot a=x\cdot a(x)\;\mathrm{mod}\;f(x),3\cdot a=(x+1)\cdot a(x)\;\mathrm{mod}\;f(x)=2\cdot a⊕a,\\ \; & 4\cdot a=x^{2}\cdot a\left(x\right)\;\mathrm{mod}\;f\left(x\right)=2^{2}\cdot a,5\cdot a=\left(x^{2}+1\right)\cdot a\left(x\right)\;\mathrm{mod}\;f\left(x\right)=2^{2}\cdot a⊕a,\\ \; & 6\cdot a=\left(x^{2}+x\right)\cdot a\left(x\right)\;\mathrm{mod}\;f\left(x\right)=2^{2}\cdot a⊕2\cdot a,\\ \; & 7\cdot a=\left(x^{2}+x+1\right)\cdot a\left(x\right)\;\mathrm{mod}\;f\left(x\right)=2^{2}\cdot a⊕2\cdot a⊕a,\\ \; & 8\cdot a=x^{3}\cdot a\left(x\right)\;\mathrm{mod}\;f\left(x\right)=2^{3}\cdot a,...\\ \; & 2^{2}\cdot a=2\cdot 2\cdot a=x^{2}\cdot a\left(x\right)\;\mathrm{mod}\;f\left(x\right)=4\cdot a,2\cdot 3\cdot a=x\left(x+1\right)\cdot a\left(x\right)\;\mathrm{mod}\;f\left(x\right)=6\cdot a,...\\ \; & 3^{2}\cdot a={\left(x+1\right)}^{2}\cdot a\left(x\right)\;\mathrm{mod}\;f\left(x\right)=\left(x^{2}+1\right)\cdot a\left(x\right)\;\mathrm{mod}\;f\left(x\right)=5\cdot a,... \end{array}$$

**Sum of Even–Mansour Construction (SoEM) [14].** SoEM introduced by Chen et al. is a provably secure pseudorandom function in the classical security model. It is built by the XOR of two distinct instances of the Even–Mansour cipher. The specification of SoEM is shown as follows. Let $P_{1}$ and $P_{2}$ be two public *n*-bit permutations. Let $K_{1}$ and $K_{2}$ be two *n*-bit keys. For a plaintext *M* and the corresponding ciphertext *C*, SoEM: ${\left\{0,1\right\}}^{2n}\times {\left\{0,1\right\}}^{n}\to {\left\{0,1\right\}}^{n}$ can be expressed as:

$$\begin{array}{c} C=SoEM_{K_{1},K_{2}}^{P_{1},P_{2}}\left(M\right)=P_{1}\left(M⊕K_{1}\right)⊕K_{1}⊕P_{2}\left(M⊕K_{2}\right)⊕K_{2}. \end{array}$$

SoEM can be divided into three variants, SoEM1, SoEM21, and SoEM22, according to the number of underlying permutations and keys. SoEM1, SoEM21, and SoEM22 are respectively shown as follows.

SoEM1: The permutations used in the two instances are identical (two instances utilize the same permutation), i.e., $P_{1}=P_{2}=P$. Then SoEM1: ${\left\{0,1\right\}}^{2n}\times {\left\{0,1\right\}}^{n}\to {\left\{0,1\right\}}^{n}$ can be expressed as:

$$\begin{array}{c} C=SoEM1_{K_{1},K_{2}}^{P}\left(M\right)=P\left(M⊕K_{1}\right)⊕K_{1}⊕P\left(M⊕K_{2}\right)⊕K_{2}. \end{array}$$

Note that, in this case, it makes no sense to subdivide again as the same key will make SoEM1 zero.

SoEM21: The permutations used in the two instances are independent but keys used in the two instances are identical, i.e., $K_{1}=K_{2}=K$. Then SoEM21: ${\left\{0,1\right\}}^{n}\times {\left\{0,1\right\}}^{n}\to {\left\{0,1\right\}}^{n}$ can be expressed as:

$$\begin{array}{c} C=SoEM21_{K}^{P_{1},P_{2}}\left(M\right)=P_{1}\left(M⊕K\right)⊕P_{2}\left(M⊕K\right)⊕K. \end{array}$$

SoEM22: The permutations used in the two instances are independent and keys used in the two instances are independent, i.e., SoEM22 is SoEM. Then SoEM22: ${\left\{0,1\right\}}^{2n}\times {\left\{0,1\right\}}^{n}\to {\left\{0,1\right\}}^{n}$ can be expressed as:

$$\begin{array}{c} C=SoEM22_{K_{1},K_{2}}^{P_{1},P_{2}}\left(M\right)=P_{1}\left(M⊕K_{1}\right)⊕K_{1}⊕P_{2}\left(M⊕K_{2}\right)⊕K_{2}. \end{array}$$

## 3. Quantum Algorithms

This section presents brief descriptions of Simon’s algorithm [3], Grover’s algorithm [2], and the Grover-meet-Simon algorithm [13].

### 3.1. Simon’s Algorithm

Simon’s algorithm [3] is an algorithm that specializes in solving period finding problem efficiently. The period finding problem is called Simon’s problem which is described as follows:

**Period Finding Problem.** Given a boolean function $f:{\left\{0,1\right\}}^{n}\to {\left\{0,1\right\}}^{n}$, assume that there exists $s\in {\left\{0,1\right\}}^{n}\setminus \left\{0^{n}\right\}$, for any $x\neq y\in {\left\{0,1\right\}}^{n}$, such that f(x)=f(y)⇔x⊕y=s. The goal is to find the period *s*.

In the classical algorithm, people solve this problem by searching and finding collisions. The optimal time complexity is $O(2^{n/2})$. While, in the quantum algorithm, by Simon’s algorithm, it can be solved in a polynomial time of *n* (i.e., O(n) quantum query complexity and O(n) qubits memory complexity). The details of Simon’s algorithm are not introduced here. We just need to know that the period finding problem can be solved by Simon’s algorithm with O(n) quantum query complexity and O(n) qubits memory complexity.

### 3.2. Grover’s Algorithm

Grover’s algorithm [2] is a quantum search algorithm that specializes in solving a search problem efficiently. The search problem is described as follows:

**The Search Problem.** Given a function $g:{\left\{0,1\right\}}^{n}\to \left\{0,1\right\}$, if $x\in {\left\{0,1\right\}}^{n}$ is a solution of the search problem, then g(x)=1, otherwise g(x)=0. The goal is to find the solution *x*.

In the classical algorithm, people solve this problem by searching this solution. The time complexity is $O(2^{n})$. While, in the quantum algorithm, by Grover’s algorithm, it can be solved in $O(2^{n/2})$ quantum query complexity and O(n) qubits memory complexity. Grover’s search algorithm improves search complexity exponentially. The details of Grover’s algorithm are not introduced here.

### 3.3. Grover-Meet-Simon Algorithm

The Grover-meet-Simon algorithm [13] is a quantum asymmetric search of a period algorithm. It combined Grover’s search algorithm with Simon’s algorithm to recover keys. The asymmetric search of a period problem is described as follows:

**Grover-meet-Simon Problem.** Let m,n,l be three positive integers, $U\subseteq {\left\{0,1\right\}}^{m}$ be a finite set, and $f:{\left\{0,1\right\}}^{m}\times {\left\{0,1\right\}}^{n}\to {\left\{0,1\right\}}^{l}$ be a function which meets that 1) if u∈U, then f(u,·) is a period function with period $s_{u}$; 2) if u∉U, then f(u,·) is an aperiodic function. The goal is to find the search-period pair $\left(u,s_{u}\right)$.

The idea of settling the Grover-meet-Simon problem is to first search u∈U by Grover’s algorithm and then check whether f(u,·) is a period function or not by Simon’s algorithm. If f(u,·) is a period function with period $s_{u}$, then $\left(u,s_{u}\right)$ is what we need. Therefore, in the quantum algorithm, the Grover-meet-Simon problem can be solved in $O\left(n\right)\times O\left(2^{n/2}\right)=O\left(n\cdot 2^{n/2}\right)$ quantum query complexity and $O\left(n\right)\times O\left(n\right)=O\left(n^{2}\right)$ qubits memory complexity. The details of the Grover-meet-Simon algorithm are not introduced here.

## 4. Quantum Attacks against SoEM with Linear Key Schedules

### 4.1. Quantum Attacks against SoEM21

Shinagawa and Iwata left it as an open problem for the analysis of the security of the following construction [27]:

$$\begin{array}{c} C=SoEM21_{K}^{P_{1},P_{2}}\left(M\right)=P_{1}\left(M⊕K\right)⊕K⊕P_{2}\left(M⊕2\cdot K\right)⊕2\cdot K. \end{array}$$

In particular, if $P_{1}=P_{2}=P$, then SoEM21 degrades to SoEM11, i.e.,

$$\begin{array}{c} C=SoEM11_{K}^{P}\left(M\right)=P\left(M⊕K\right)⊕K⊕P\left(M⊕2\cdot K\right)⊕2\cdot K. \end{array}$$

For the above SoEM11 and SoEM21 constructions, we present quantum attacks in Theorems 1 and 2.

**Theorem** **1.** *There exists a quantum key recovery attack against SoEM11 in O(n) quantum query complexity and O(n) qubits memory complexity.*

**Proof.**  Our proof utilizes Simon’s algorithm. By careful observation of SoEM11, we find that SoEM11 itself is a period function with period 3·K. To be specific, let $f:{\left\{0,1\right\}}^{n}\to {\left\{0,1\right\}}^{n}$ be a function, which is defined as:

$$\begin{array}{c} f\left(x\right)=SoEM11_{K}^{P}\left(x\right)=P\left(x⊕K\right)⊕P\left(x⊕2\cdot K\right)⊕3\cdot K. \end{array}$$

It follows that,

$$\begin{array}{cc} f(x⊕3\cdot K) & =P(x⊕3\cdot K⊕K)⊕P(x⊕3\cdot K⊕2\cdot K)⊕3\cdot \;K\\ \; & =P(x⊕2\cdot K)⊕P(x⊕K)⊕3\cdot K=f(x), \end{array}$$

where K⊕2·K=3·K, K⊕3·K=2·K, and 3·K⊕2·K=K.

Therefore, *f* is a period function with period 3·K. Then, 3·K can be derived in O(n) quantum queries and O(n) qubits memory complexity to *f* by Simon’s algorithm. It follows that, K=3·K/3 can be recovered. □

**Theorem** **2.** *There exists a quantum key recovery attack against SoEM21 in $O(2^{n/2})$ quantum query complexity and O(n) qubits memory complexity.*

**Proof.**  Our proof utilizes Grover’s algorithm. First, we construct a new function $f:{\left\{0,1\right\}}^{n}\times {\left\{0,1\right\}}^{n}\to {\left\{0,1\right\}}^{n}$ as:

$$\begin{array}{cc} f(k,x) & =SoEM21_{K}^{P_{1},P_{2}}\left(x\right)⊕P_{1}\left(x\right)⊕P_{2}\left(x⊕k\right)\\ \; & =P_{1}\left(x⊕K\right)⊕K⊕P_{2}\left(x⊕2\cdot K\right)⊕2\cdot K⊕P_{1}\left(x\right)⊕P_{2}\left(x⊕k\right)\\ \; & =P_{1}\left(x⊕K\right)⊕P_{1}\left(x\right)⊕P_{2}\left(x⊕2\cdot K\right)⊕P_{2}\left(x⊕k\right)⊕3\cdot K. \end{array}$$

By careful observation, we find that if k=3·K, then f(3·K,·) is a period function with period *K*, as:

$$\begin{array}{cc} f(3\cdot K,x⊕K)= & P_{1}\left(x⊕K⊕K\right)⊕P_{1}\left(x⊕K\right)\\ \; & ⊕P_{2}\left(x⊕K⊕2\cdot K\right)⊕P_{2}\left(x⊕K⊕3\cdot K\right)⊕3\cdot \;K\\ = & P_{1}\left(x\right)⊕P_{1}\left(x⊕K\right)⊕P_{2}\left(x⊕3\cdot K\right)⊕P_{2}\left(x⊕2\cdot K\right)⊕3\cdot \;K\\ = & f(3\cdot K,x). \end{array}$$

Therefore, we first search k=3·K by Grover’s algorithm and then verify whether f(3·K,·) is a period function with a period K=3·K/3 or not. Therefore, *K* can be derived in a $O(2^{n/2})$ quantum queries to *f* and O(n) qubits memory complexity by Grover’s algorithm. □

### 4.2. Quantum Attacks against SoEM with Linear Key Schedules

We consider a generalized construction of SoEM21 with linear key schedules and rename it as SoEM21L, i.e.,

$$\begin{array}{c} C=SoEM21L_{K}^{P_{1},P_{2}}\left(M\right)=P_{1}\left(M⊕a\cdot K\right)⊕P_{2}\left(M⊕b\cdot K\right)⊕c\cdot K, \end{array}$$

where a,b,c are three integers and (a,b,c)≠(0,0,0).

In particular, if $P_{1}=P_{2}=P$ and a≠b, then SoEM21L degrades to SoEM11L, i.e.,

$$\begin{array}{c} C=SoEM11L_{K}^{P}\left(M\right)=P\left(M⊕a\cdot K\right)⊕K⊕P\left(M⊕b\cdot K\right)⊕c\cdot K. \end{array}$$

For the above SoEM21L and SoEM11L constructions, we present quantum attacks in Theorems 3 and 4.

**Theorem** **3.** *There exists a quantum key recovery attack against SoEM11L in O(n) quantum query complexity and O(n) qubits memory complexity.*

**Proof.**  Our proof utilizes Simon’s algorithm. By careful observation of SoEM11L, we find that SoEM11L itself is a period function with period (a⊕b)·K. To be specific, let $f:{\left\{0,1\right\}}^{n}\to {\left\{0,1\right\}}^{n}$ be a function, which is defined as:

$$\begin{array}{c} f\left(x\right)=SoEM11L_{K}^{P}\left(x\right)=P\left(x⊕a\cdot K\right)⊕P\left(x⊕b\cdot K\right)⊕c\cdot K. \end{array}$$

It follows that,

$$\begin{array}{cc} f(x⊕(a⊕b)\cdot K) & =P(x⊕(a⊕b)\cdot K⊕a\cdot K)⊕P(x⊕(a⊕b)\cdot b⊕2\cdot K)⊕c\cdot \;K\\ \; & =P(x⊕b\cdot K)⊕P(x⊕a\cdot K)⊕c\cdot K=f(x), \end{array}$$

where a·K⊕b·K=(a⊕b)·K, a·K⊕(a⊕b)·K=b·K, and (a⊕b)·K⊕b·K=a·K.

Therefore, *f* is a period function with period (a⊕b)·K. Then, (a⊕b)·K can be derived in polynomial time of *n* (O(n) qubits and O(n) quantum oracle queries to *f*) by Simon’s algorithm. It follows that, K=(a⊕b)·K/(a⊕b) can be recovered. □

**Theorem** **4.** *There exists a quantum key recovery attack against SoEM21L in $O(2^{n/2})$ quantum query complexity and O(n) qubits memory complexity.*

**Proof.**  Our proof utilizes Grover’s algorithm. We construct a new function $f:{\left\{0,1\right\}}^{n}\times {\left\{0,1\right\}}^{n}\to {\left\{0,1\right\}}^{n}$ as:

$$\begin{array}{cc} f(k,x) & =SoEM21L_{K}^{P_{1},P_{2}}\left(x\right)⊕P_{1}\left(x\right)⊕P_{2}\left(x⊕k\right)\\ \; & =P_{1}\left(x⊕a\cdot K\right)⊕P_{2}\left(x⊕b\cdot K\right)⊕P_{1}\left(x\right)⊕P_{2}\left(x⊕k\right)⊕c\cdot K. \end{array}$$

By careful observation, we find that if k=(a⊕b)·K, then *f* is a period function with period a·K, i.e.,

$$\begin{array}{cc} f((a⊕b)\cdot K,x⊕a\cdot K)= & P_{1}\left(x⊕a\cdot K⊕a\cdot K\right)⊕P_{2}\left(x⊕a\cdot K⊕b\cdot K\right)\\ \; & ⊕P_{1}\left(x⊕a\cdot K\right)⊕P_{2}\left(x⊕a\cdot K⊕\left(a⊕b\right)\cdot K\right)⊕c\cdot \;K\\ = & P_{1}\left(x\right)⊕P_{2}\left(x⊕\left(a⊕b\right)\cdot K\right)\\ \; & ⊕P_{1}\left(x⊕a\cdot K\right)⊕P_{2}\left(x⊕b\cdot K\right)⊕c\cdot \;K\\ = & f((a⊕b)\cdot K,x). \end{array}$$

Therefore, for any SoEM21L, we first search k=(a⊕b)·K by Grover’s algorithm and then verify whether f((a⊕b)·K,·) is a period function with a period a·K or not. It follows that *K* can be derived in a $O(2^{n/2})$ quantum queries to *f* and O(n) qubits memory complexity by Grover’s algorithm. □

## 5. Generalizations and Attacks

Inspired by linear key schedules, this section considers natural generalizations of SoEM1, SoEM21, and SoEM22, and presents quantum key recovery attacks against these constructions.

### 5.1. Generalizations

We define SoEM1sL, SoEMs1L, and SoEMssL as natural generalizations of SoEM1, SoEM21, and SoEM22 with linear key schedules, respectively. The constructions of them are respectively shown as follows.

Let s≥2 and $\left(a_{1},a_{2},\cdots ,a_{s}\right)\neq \left(0,0,\cdots ,0\right)$ be integers. Let $P_{1},\cdots ,P_{s}$ be *s* public *n*-bit permutations, $K_{1},\cdots ,K_{s}$ be *sn*-bit keys, *M* be a plaintext, and *C* be a ciphertext, then SoEMssL (SoEM with *s* permutations and *s* linear keys) is defined as:

$$\begin{array}{cc} C & =SoEMssL_{K_{1},\cdots ,K_{s}}^{P_{1},\cdots ,P_{s}}\left(M\right)\\ \; & =P_{1}\left(M⊕a_{1}\cdot K_{1}\right)⊕a_{1}\cdot K_{1}⊕\cdots ⊕P_{s}\left(M⊕a_{s}\cdot K_{s}\right)⊕a_{s}\cdot K_{s}. \end{array}$$

If $P_{1}=\cdots =P_{s}=P$, then SoEMssL will degrade to SoEM1sL which is defined as:

$$\begin{array}{cc} C & =SoEM1sL_{K_{1},\cdots ,K_{s}}^{P}\left(M\right)\\ \; & =P\left(M⊕a_{1}\cdot K_{1}\right)⊕a_{1}\cdot K_{1}⊕\cdots ⊕P\left(M⊕a_{s}\cdot K_{s}\right)⊕a_{s}\cdot K_{s}. \end{array}$$

If $K_{1}=\cdots =K_{s}=K$, then SoEMssL will degrade to SoEMs1L, which is defined as:

$$\begin{array}{cc} C & =SoEMs1L_{K}^{P_{1},\cdots ,P_{s}}\left(M\right)\\ \; & =P_{1}\left(M⊕a_{1}\cdot K\right)⊕\cdots ⊕P_{s}\left(M⊕a_{s}\cdot K\right)⊕a_{s+1}\cdot K, \end{array}$$

where $a_{s+1}$ is an arbitrary integer.

If $P_{1}=\cdots =P_{s}=P$, $K_{1}=\cdots =K_{s}=K$, and $a_{1}\neq a_{2}\neq \cdots \neq a_{s}$, then SoEMssL will degrade to SoEM11L, which is defined as:

$$\begin{array}{cc} C & =SoEM11L_{K}^{P}\left(M\right)\\ \; & =P\left(M⊕a_{1}\cdot K\right)⊕\cdots ⊕P\left(M⊕a_{s}\cdot K\right)⊕a_{s+1}\cdot K, \end{array}$$

where $a_{s+1}$ is an arbitrary integer.

### 5.2. Quantum Key Recovery Attacks

**Theorem** **5.** *There exists a quantum key recovery attack against SoEM1sL that obtains the secret key $K_{1},\cdots ,K_{s}$ in $O(n^{2}+sn)$ qubits and $O(sn\cdot 2^{(s-1)n/2})$ quantum queries.*

**Proof.**  Our attack is based on the Grover-meet-Simon algorithm and is similar with the attack against SoEMss [27]. We consider two functions $g:{\left\{0,1\right\}}^{(s-1)n}\times {\left\{0,1\right\}}^{n}\to {\left\{0,1\right\}}^{n}$ and $f:{\left\{0,1\right\}}^{(s-1)n}\times {\left\{0,1\right\}}^{n}\to {\left\{0,1\right\}}^{n}$, which are defined as follows.

$$\begin{array}{cc} g(k_{2},\cdots ,k_{s},x)= & P\left(x\right)⊕P\left(x⊕a_{2}\cdot k_{2}\right)⊕\cdots ⊕P\left(x⊕a_{s}\cdot k_{s}\right),\\ f(k_{2},\cdots ,k_{s},x)= & SoEM1sL_{K_{1},\cdots ,K_{s}}^{P}\left(x\right)⊕g\left(k_{2},\cdots ,k_{s},x\right)\\ = & P\left(x⊕a_{1}\cdot K_{1}\right)⊕a_{1}\cdot K_{1}⊕\cdots ⊕P\left(x⊕a_{s}\cdot K_{s}\right)⊕a_{s}\cdot \;K_{s}\\ \; & ⊕P\left(x\right)⊕P\left(x⊕a_{2}\cdot k_{2}\right)⊕\cdots ⊕P\left(x⊕a_{s}\cdot k_{s}\right). \end{array}$$

If $\left(k_{2},\cdots ,k_{s}\right)=\left(K_{2},\cdots ,K_{s}\right)$, then $f\left(K_{2},\cdots ,K_{s},x\right)=P\left(x⊕a_{1}\cdot K_{1}\right)⊕a_{1}\cdot K_{1}⊕\cdots ⊕a_{s}\cdot K_{s}⊕P\left(x\right)$ and $f(K_{2},\cdots ,K_{s},x)$ is a period function with period $a_{1}\cdot K_{1}$. Therefore, by Simon’s algorithm, we can obtain the period $a_{1}\cdot K_{1}$. It follows that we recover $K_{1}=a_{1}\cdot K_{1}/a_{1}$.

Then we utilize Grover’s algorithm to recover $K_{2},\cdots ,K_{s}$. Similar with FX construction and SoEM22, we utilize the Grover-meet-Simon algorithm to find the value of $\left(k_{2},\cdots ,k_{s}\right)$ that makes $f(k_{2},\cdots ,k_{s},x)$ period. If we find a period function, then, at this point, $\left(k_{2},\cdots ,k_{s}\right)$ is the secret keys $\left(K_{2},\cdots ,K_{s}\right)$ that we need to recover and the period is $a_{1}\cdot K_{1}$.

Therefore, for any SoEM1sL, we can construct two functions *f* and *g*. By the Grover-meet-Simon algorithm, $\left(K_{1},K_{2},\cdots ,K_{s}\right)$ can be derived in $O(n^{2}+sn)$ qubits and $O(sn\cdot 2^{(s-1)n/2})$ quantum oracle queries to *f* and *g*. □

**Theorem** **6.** *There exists a quantum key recovery attack against SoEMs1L that obtains the secret key K in O(n) qubits and $O(2^{n/2})$ quantum oracle queries.*

**Proof.**  Our attack is based on Grover’s algorithm and is a generalization of the quantum attack against SoEM21L. We consider a function $f:{\left\{0,1\right\}}^{n}\times {\left\{0,1\right\}}^{n}\to {\left\{0,1\right\}}^{n}$, which is defined as follows.

$$\begin{array}{cc} f(k,x)= & SoEMs1L_{K}^{P_{1},\cdots ,P_{s}}\left(x\right)⊕P_{1}\left(x\right)⊕P_{2}\left(x⊕a_{2}\cdot k\right)⊕\cdots ⊕P_{s}\left(x⊕a_{s}\cdot k\right)\\ = & P_{1}\left(x⊕a_{1}\cdot K\right)⊕\cdots ⊕P_{s}\left(x⊕a_{s}\cdot K\right)⊕a_{s+1}\cdot \;K\\ \; & ⊕P_{1}\left(x\right)⊕P_{2}\left(x⊕a_{2}\cdot k\right)⊕\cdots ⊕P_{s}\left(x⊕a_{s}\cdot k\right). \end{array}$$

By careful observation, we find that if k=K, then *f* is a period function with period $a_{1}\cdot K$, i.e., $f\left(K,x⊕a_{1}\cdot K\right)=f\left(K,x\right)$.

Therefore, for any SoEMs1L, we first search k=K by Grover’s algorithm and then verify whether f(K,·) is a period function with a period $a_{1}\cdot K$ or not. It follows that *K* can be derived in the $O(2^{n/2})$ quantum queries to *f* and O(n) qubits memory complexity by Grover’s algorithm. □

**Theorem** **7.** *There exists a quantum key recovery attack against SoEMssL that recovers the secret key $K_{1},\cdots ,K_{s}$ in $O(n^{2}+sn)$ qubits and $O(sn\cdot 2^{(s-1)n/2})$ quantum queries.*

**Proof.**  Our attack is based on the Grover-meet-Simon algorithm and is similar with the attack against SoEMss [27]. We consider two functions $g:{\left\{0,1\right\}}^{(s-1)n}\times {\left\{0,1\right\}}^{n}\to {\left\{0,1\right\}}^{n}$ and $f:{\left\{0,1\right\}}^{(s-1)n}\times {\left\{0,1\right\}}^{n}\to {\left\{0,1\right\}}^{n}$, which are defined as follows:

$$\begin{array}{cc} g(k_{2},\cdots ,k_{s},x)= & P_{1}\left(x\right)⊕P_{2}\left(x⊕a_{2}\cdot k_{2}\right)⊕\cdots ⊕P_{s}\left(x⊕a_{s}\cdot k_{s}\right),\\ f(k_{2},\cdots ,k_{s},x)= & SoEM1sL_{K_{1},\cdots ,K_{s}}^{P_{1},\cdots ,P_{s}}\left(x\right)⊕g\left(K_{2},\cdots ,K_{s},x\right)\\ = & P_{1}\left(x⊕a_{1}\cdot K_{1}\right)⊕a_{1}\cdot K_{1}⊕\cdots ⊕P_{s}\left(x⊕a_{s}\cdot K_{s}\right)⊕a_{s}\cdot \;K_{s}\\ \; & ⊕P_{1}\left(x\right)⊕P_{2}\left(x⊕a_{2}\cdot k_{2}\right)⊕\cdots ⊕P_{s}\left(x⊕a_{s}\cdot k_{s}\right). \end{array}$$

If $\left(k_{2},\cdots ,k_{s}\right)=\left(K_{2},\cdots ,K_{s}\right)$, then $f\left(K_{2},\cdots ,K_{s},x\right)=P_{1}\left(x⊕a_{1}\cdot K_{1}\right)⊕a_{1}\cdot K_{1}⊕\cdots ⊕a_{s}\cdot K_{s}⊕P_{1}\left(x\right)$ and $f(K_{2},\cdots ,K_{s},x)$ is a period function with period $a_{1}\cdot K_{1}$. Therefore, by Simon’s algorithm, we can obtain the period $a_{1}\cdot K_{1}$. It follows that we recover $K_{1}\;=\;a_{1}\;\cdot \;K_{1}/a_{1}$.

Then we utilize Grover’s algorithm to recover $K_{2},\cdots ,K_{s}$. Similar with FX construction and SoEM22, we utilize the Grover-meet-Simon algorithm to find the value of $\left(k_{2},\cdots ,k_{s}\right)$ that makes $f(k_{2},\cdots ,k_{s},x)$ period. If we find a period function, then $\left(k_{2},\cdots ,k_{s}\right)$ is the secret keys $\left(K_{2},\cdots ,K_{s}\right)$ and the period is $a_{1}\cdot K_{1}$.

Therefore, for any SoEMssL, we can construct two functions *f* and *g*. By the Grover-meet-Simon algorithm, $\left(K_{1},K_{2},\cdots ,K_{s}\right)$ can be derived in $O(n^{2}+sn)$ qubits and $O(sn\cdot 2^{(s-1)n/2})$ quantum oracle queries to *f* and *g*. □

## 6. Conclusions and Future Works

Shinagawa and Iwata left two open problems in their paper and this paper settles one of them. For variants of SoEM, we set up a generalized construction with linear key schedules and found their quantum attacks. This paper also considered natural generalizations of SoEM with linear key schedules and presents quantum key recovery attacks. For non-linear variants, quantum attacks could recover the intermediate state, and then use some new techniques to recover the key. This paper focuses on the intuitive consequences of quantum attacks, so there is no discussion of non-linear variants. Therefore, one of the future works is to discuss the quantum attacks for non-linear variants and to try make quantum attacks for other symmetric cryptographic schemes. Other future works is to settle another open problem.

## Data Availability

The data used to support the findings of the study are available within the article.
